# Assessment of organizational carbon footprints in a denim-washing company: a systematic approach to indirect non-energy emissions

**DOI:** 10.1007/s11356-024-33640-z

**Published:** 2024-05-14

**Authors:** Hülya Aykaç Özen, Bahar Vayiç, Semra Çoruh

**Affiliations:** Department of Environmental Engineering, Ondokuz Mayıs University, Samsun, Turkey

**Keywords:** Carbon footprint, Company, Direct emissions, Indirect energy–related emissions, Indirect non-energy–related emissions

## Abstract

As stated in the 2016 Paris Agreement, concerns about global climate change and carbon emissions have increased, and organizations, in particular, have embarked on an annual measurement process to estimate their contribution to global climate change. Carbon footprint, one of the measurement methods, is a widely applied tool to assess the environmental impact of organizations. This study presents a real case study of a denim-washing company’s activities based on ISO standard calculation methods of greenhouse gas emissions. Accordingly, the annual carbon footprint of the denim-washing company was 2482.09 tCO_2_e for the year 2021 in total for the overall carbon footprint. Direct emission was calculated at 1575.75 tCO_2_e, indirect energy–related emission at 798.09 tCO_2_e, and indirect non-energy–related emission at 108.25 tCO_2_e. The highest CO_2_ emissions are related to heating from greenhouse gas direct emission sources, followed by purchased electricity consumption, and the lowest CO_2_ emissions are related to fire–CO_2_ tube storage. In conclusion, this study is particular in that it analyzes not only the specific processes of a denim-washing company but also the overall organizational carbon footprint calculation, assesses the importance of indirect non-energy in the total carbon footprint, and evaluates the calculation findings with sector-specific mitigation strategies.

## Introduction

Climate change is one of the most significant global challenges that can no longer be ignored (Chang et al. 2015; Padrón et al. 2020; Xie et al. 2020; Ye et al. 2013; Zhang et al. 2013). The 21st session of the Conference of the Parties (COP21), held in Paris to combat climate change and discuss activities and investments for a low-carbon and sustainable future, resulted in the Paris Agreement signed by 197 countries. The Paris Agreement is an international agreement in which countries recognize the need for joint action to prevent the climate crisis. It draws attention to the importance of identifying and minimizing the losses and damages caused by climate change and limiting the increase in the global average surface temperature to 2 °C compared to the pre-industrial period and keeping it below 1.5 °C if possible to prevent the climate crisis (EU 2016). In parallel with the temperature change emphasized in the Paris Agreement, data from the Intergovernmental Panel on Climate Change (IPCC) highlights that a 1.5 °C warming would be relatively safer than a 2 °C warming. According to the IPCC, the risk of flooding, expected to increase by 100% with a 1.5 °C increase in average surface temperature, would reach 170% with a 2 °C warming. From this perspective, it is imperative, especially for countries that have signed the Paris Agreement, to develop green policies and strategies to reduce the contribution of their industries to climate change. Regardless of the sector, low-carbon/carbon–neutral products and services are rapidly gaining popularity and preference among customers and investors (Dawkins and Fraas 2011; Foran et al. 2005; Scipioni et al. 2012; Sundarakani et al. 2010). Establishing greenhouse gas emissions inventories for climate change adjustment conditions and action plans for organizations is the first step in developing a green policy. Organizations, therefore, need to use suitable tools for evaluating their impact on the climate (Radonjič and Tompa 2018). The carbon footprint approach is one of the ways companies use, considering that climate change is the most critical issue based on international standards (Peters 2010; Ruževičius and Dapkus 2018). The organizational carbon footprint is calculated by gathering information about its consumption and converting it to equal CO2 emissions directly and indirectly caused by various activities (Weidema et al. 2008; Wiedmann and Minx 2008).

Companies can calculate their carbon footprint at individual, product, and organizational levels. An individual carbon footprint is the amount of carbon dioxide emitted from clothing, housing, diet, and transportation. A product’s carbon footprint calculates the greenhouse gas emissions that occur during its whole life, from the extraction of materials and their production to use, disposal, recycling, or reuse. An organization’s carbon footprint calculates the greenhouse gas emissions from all of its operations, including the energy required to operate buildings, manufacturing facilities, company transportation, and business travel of employees. In other words, personal and product emissions cover the activities of the whole product’s lifecycle, while organization CFP quantifies the emissions from an organization’s activities. Currently, numerous initiatives, guidelines, and calculation methods exist for measuring greenhouse gas emissions at the organizational level. Publicly Available Specification (PAS) guidelines published by the Institute of British Standards, the ISO 14064 International Organization for Standardization, the Greenhouse Protocol developed by the World Resources Institute, and the World Business Council for Sustainable Development are the most common methods for calculating the environmental impact and carbon footprint (Gao et al. 2014; Karalis and Kanakoudis 2023). The most popular of these techniques is the ISO 14064:^2018 ^standard on Greenhouse gases—Part 1: Specification with guidance at the organization level for quantifying and reporting greenhouse gas emissions and removals (ISO-14064–1 2018). An organization’s carbon footprint can be separated into different categories according to ISO 14064–1. This includes emissions that the organization directly produces and indirect energy–related emissions and non-energy–related emissions depending on the organization’s boundaries (Harangozo and Szigeti 2017). Direct emissions come from sources owned or managed by the organization or directly emit greenhouse gases inside the organization’s boundaries. These sources can be stationary or mobile. Indirect energy–related emissions include greenhouse gas emissions from fuel combustion, which are involved in energy generation and related services such as electricity, heat, steam, refrigeration, and compressed air. Note that direct and indirect energy–related emissions calculate emissions from sources originating from the organization, whereas indirect non-energy–related emissions are mainly produced outside the organization’s borders. A data source must be provided for indirect non-energy–related emissions to ensure reliability and comparability, as well as the effective implementation of a carbon footprint indicator (Alvarez and Rubio 2015). This was supported by several authors who noted that indirect non-energy–related emissions frequently have the most significant impact on greenhouse gas emissions for an organization, and therefore, underestimating them would typically result in significantly higher greenhouse gas emissions (Matthews et al. 2008). Indirect non-energy–related emission is divided into sub-categories. These categories include emissions from vehicles for hire, services used by an organization for the transport of persons and goods (rail, maritime, air, and road), sources not owned or controlled by the organization, and any type of goods purchased by the reporting organization. Consequently, it is the responsibility of the organization to define the content of these specific categories.

Several companies and organizations have taken the initiative to determine their own carbon footprints over the past 10 years. The risk associated with global warming has recently been calculated by various organizations, including universities, the service sector, the cement industry, the gas refinery industry, the wine industry, the automotive industry, telecommunications companies, and logistics services (Alvarez and Rubio 2015; Cagiao et al. 2011; Eslamidoost et al. 2022; Karalis and Kanakoudis 2023; Lee and Cheong 2011; Radonjič and Tompa 2018; Robinson et al. 2018; Ruževičius and Dapkus 2018; Saenz et al. 2016). The low-carbon environmental protection of the whole industry has a significant impact on climate change. The textile industry, a primary global export sector and a crucial hub for production and processing, has to deal with serious environmental issues (Khan and Malik 2014; Kishor et al. 2020; Sivaram et al. 2019; Zhou et al. 2022). Although there are several relevant studies on greenhouse gas emissions in the textile sector, they tend to focus on specific products and production methods. According to the comprehensive literature search, the characteristics of organizational carbon footprint applied to textile industries have only been briefly discussed in a few research studies. The greenhouse gas emissions from China’s textile sector are thoroughly examined, along with the emission characteristics, by Huang et al. The findings indicate that coal consumption is the most significant cause of greenhouse gas emissions in China’s textile industry, accounting for 80% of all greenhouse gases from primary energy sources. The consumption of electricity is the second-largest source of greenhouse gas emissions (Huang et al. 2017). An examination of direct and indirect emissions in Chinese textiles was done by Yan and colleagues. About 87% of the overall carbon footprint is contributed by indirect carbon footprint, the main source of which is energy use, whereas the direct industrial carbon footprint only makes up about 13% of this total (Yan et al. 2016).

As stated before, the literature review reveals a few associated research efforts related to calculating the carbon footprint and reducing greenhouse gas emissions for the textile sector; however, most focused on particular processes rather than examining an organization as a whole. Furthermore, a calculation based only on direct emissions from a company’s own operations leads to an incorrect assessment of the company’s overall greenhouse gas emissions. This means that indirect non-energy–related emissions often represent a considerable greenhouse gas impact on a company, and ignoring them often leads to a significant underestimation of greenhouse gas emissions. To our knowledge, no study conducts a detailed indirect carbon footprint assessment of a denim-washing company at the organizational level. To fill this gap, this paper aims to identify the emission hotspots of an organization in the denim-washing sector based on ISO standard calculation methods of greenhouse gas emissions, estimate its carbon footprint to reduce its impact, and suggest alternatives. The three categories are considered to calculate the organization’s carbon footprint in this study. The first category refers to direct emissions from the company’s stationary and mobile fuel consumption, the second category is indirect emissions from electric power consumption, and the last is emissions from non-company-owned vehicles, employee commuting, waste disposal, and water consumption. The main contributions of this study are to comprehensively assess the direct and indirect energy–related and indirect non-energy–related categories of the organizational carbon footprint of the denim-washing company to contribute to the knowledge of sector-specific indirect greenhouse gas emissions and identify sector-specific mitigation strategies.

## Materials and method

### Study area and organizational boundaries

**Fig. 1:**
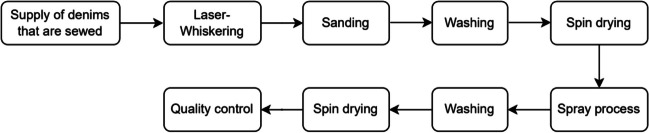
The overall flow chart of the denim-washing company

The company operates in denim washing, and their average production amount in 2021 is 202.41 t. It employs 55 people, working 8 h a day and 300 days a year. The overall flow chart of the company is presented in Fig. 1, and its detailed description is as follows: The denim product undergoes a whiskering process to create straight or wavy lines of varying lengths according to the desired model. The sanding process is then initiated. Subsequently, the products undergo pre-washing to ensure they are clean. The fabric undergoes a stone-washing process to create the desired effect on its surface. This process involves washing the fabric with stone enzymes and pumice stones, which are reused for subsequent washes. Once the stone-washing process is complete, the stones are rinsed and sent for spinning and drying. Following this stage, various processes are applied to the denim and garments to achieve different effects and features. To lighten the ground color of the denim, a diluted chemical is applied to the trousers using a brush. The abrasion process can also be carried out using a laser. The products are then sent to the washing department to clean the denims. The washing process involves the use of a dispersing agent and sizing enzyme. Finally, the products are rinsed with water and dried. Quality control checks are then carried out once all processes are completed.

**Fig. 2:**
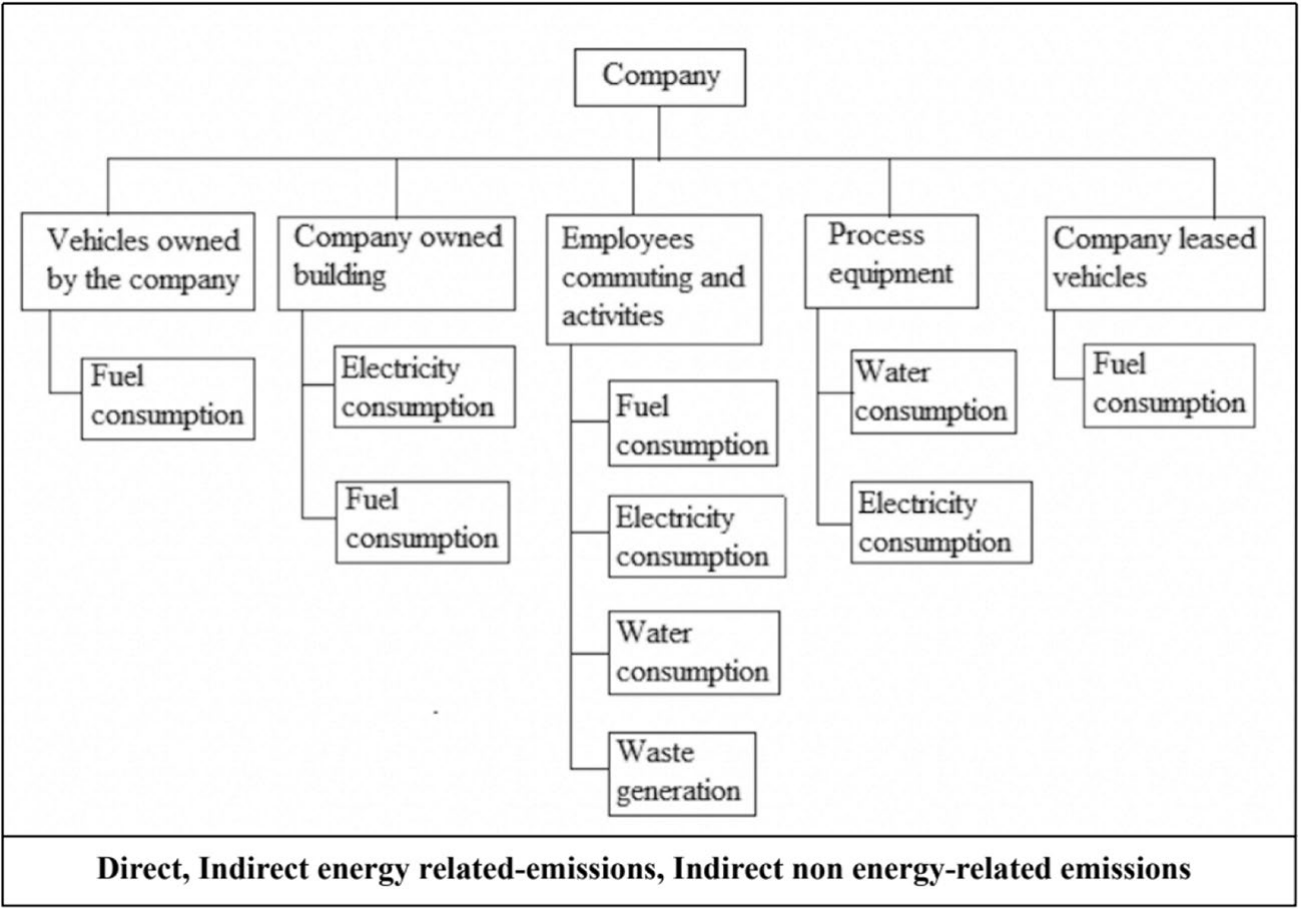
Organizational chart for the denim-washing company under investigation

System boundaries were defined to calculate direct and indirect greenhouse gas emissions associated with the organization’s operations. The boundaries were determined from the gate-to-gate approach, starting with the product entering the plant until the washing process was completed and the product left the plant. The organizational boundaries based on the company’s activities are displayed in Fig. 2.

In this study, the system boundary is analyzed in three categories in order to calculate carbon footprint Protocol 2004. Direct emissions include stationary combustion emissions from natural gas used by the company, mobile combustion emissions from company-owned vehicles, emissions from using generators and forklifts, and emissions from using CO_2_ storage in fire tubes. Indirect energy–related emissions include emissions from the use of electricity purchased by the company. Other indirect non-energy–related emissions include water use, waste disposal, and employees commuting as well as emissions from cars that are not company-owned.

### Data collection

All significant greenhouse gas emission sources were first identified to prepare the organization’s greenhouse gas emission inventory. The activity data related to emissions for 2021 was obtained from the company’s own departments and is listed in Table 1. The company’s fuel consumption and purchase data for heating and transportation for business travel were used to collect information about direct greenhouse gas emissions categorized in the direct emission category. The company’s own building is heated with natural gas. The calculation methodology specified in the Department for Environment, Food and Rural Affairs (DEFRA) was used to evaluate greenhouse gas emissions caused by natural gas (DEFRA 2021). In order to determine transport greenhouse gas emissions, the total distance traveled, fuel type, and technical specifications of car manufacturers were used, which provided the company’s own documentation. Also, fuel consumption data for the diesel of the generator and forklift were obtained from the company’s records. The tool developed by the DEFRA was used to determine greenhouse gas emissions from mobile combustion and fuel consumption.

**Table 1:** Data on the activities of a denim-washing company in 2021

	Activity	Data	Unit	Source
Direct emissions	Heating	612.79	ton	Natural gas bills
	Use of factory-owned car for business travel	78,910	km	Invoices
	The use of the generator	400	lt	Running charts
	The use of a forklift	400	lt	Running charts
	CO_2_ storage in fire tubes	35	kg	Running charts
Indirect energy–related emissions	Purchased electricity consumption	1,618,396	kWh	Electricity bills
Indirect non-energy–related emissions	Employees commuting	138,600	km	Questionnaire
	Hired vehicle	33,088	km	Invoices
	Consumption of water	118,000	m^3^	Water bills
	Generation of waste	142,597	kg	Running charts

Activity data related to greenhouse gas emissions classified in the indirect energy–related emission category includes electricity consumption purchased from the grid. The use of lighting, automated building processes, and electricity used for operation are all included in the energy usage. The activity data for the electricity purchase are derived from the monthly reading of electricity consumption. Greenhouse gas emission–related electricity was calculated using the national grid average emission factor that best characterizes the relevant grid. Indirect non-energy–related emissions included employees’ travel, hired vehicles, water consumption, and waste generation. Employees’ travel data in 2021 were determined from questionnaires applied through employee interviews. Total distances traveled, modes of transport, and type of cars employees travel to and from work were used to evaluate the fuel consumption. Greenhouse gas emissions related to employees’ travel are calculated on the basis of per kilometer using the DEFRA tool. Data on waste generation from the company’s activities and causing greenhouse gas emissions were gathered from running charts. The waste type and amount produced were obtained from the company’s own paperwork and records. Based on the distance traveled in 2021, the activity data for rental automobiles were computed. For this purpose, company-owned documents from business trips were used. The amount of water consumed to calculate greenhouse gas emissions caused by water consumption is obtained from monthly water consumption readings, and the water given over the network is indicated in cubic meters of water invoices. The water consumption comprises office (drinking water, cleaning, washing, vb.) and field (water for operation) activities. All indirect non-energy–related emissions were included in the calculation with the DEFRA tool.

### Carbon footprint methodology

The calculation of the company’s carbon footprint followed the ISO 14064:2018 standard on Greenhouse gases—Part 1: Specification with guidance at the organization level for quantifying and reporting greenhouse gas emissions and removals (ISO 14064–1:^2018^). The guidelines and specifications for constructing, developing, managing, and reporting greenhouse gas emission inventories at the organizational level are described in this document.

Greenhouse gas emissions from the company’s activities were determined by the relevant categories’ emission factors related to each activity data. The carbon footprint (CF) approach used in this study is based on activity data multiplied by appropriate emission factors (EFs) that calculate CO_2_e emissions or removals per unit activity using the following equations:

1 $${\text{ACF}}={\text{AD}}\times {\text{EF}}$$

2 $${\text{TCF}}={{\text{ACF}}}_{1}+{{\text{ACF}}}_{2}+...+{{\text{ACF}}}_{{\text{n}}}$$

where ACF is the carbon footprint caused by each activity, AD is the data of the activity in tons (t), EF is a standard rate of emissions per unit of activity (CO_2_e/t), and TCF is the total carbon footprint of the organization expressed in a ton of carbon dioxide equivalents (tCO_2_e).

In order to calculate carbon footprint, the conversion factors published in 2021 by the Department for Environment, Food and Rural Affairs (DEFRA) were utilized for each activity in operating boundaries. The DEFRA emission factor expresses the relationship between the amount of pollutants produced and the amount of raw material processed or burnt. The government conversion factor spreadsheets provide the values to be used for these types of conversions and detailed instructions on how to utilize them. Every year, a new set of conversion factors is produced and published, along with a document outlining the key modifications to the most recent year’s factors and a methodology outlining how the conversion factors are formed (DEFRA 2021). For purchased electricity, the national emission factor is calculated based on the total fuel consumption and net electricity generated. The Turkish Electricity Generation Corporation report states that 33.2% of natural gas, 30.9% of coal, 16.7% of hydro, 19.1% of other renewable and waste, and 0.1% of liquid fuels were delivered to the network in this region.

## Results and discussion

### Organizational carbon footprint of denim-washing company

#### Direct emissions

Natural gas consumption starts with the product entering the factory and includes the consumption of the product until the washing process is completed. This organization recorded the total natural gas consumption in 2021 as 612.79 t. DEFRA calculation methodology was used to calculate the carbon footprint resulting from the use of natural gas. Table 2 provides detailed information on the emissions produced from natural gas consumption. In order to calculate the emissions of the organization from transport, it is important to know the organization’s vehicle fleet. In 2021, the organization had 1 transporter van and 1 personal car with diesel fuel type. The transporter van was responsible for the carbon footprint with a total distance of around 56,144 km in 2021, while the diesel car accounted for about 22,766 km. Emission values were calculated by determining the appropriate emission factors from DEFRA (2021) according to the distance traveled by vehicles and specific car types owned by the institution in 2021. Emissions resulting from the use of generators, forklifts, and CO_2_ storage tanks, which are among the domestic activities, are also classified as direct emissions. In 2021, 400 l of diesel was consumed due to the activities of forklifts used to transport products from one place to another in the company. Similarly, the diesel consumption of the generators used in the company in 2021 is 400 l. The company has 7 pieces of 5 kg CO_2_ tubes to be used during a fire. In the calculation of emissions from these activities, the reference value tables of emission factors provided by DEFRA were used. Fugitive emission estimates for storage systems are taken from IPCC guidelines. Greenhouse gas emission values and carbon footprint amounts calculated for the direct emission sources of the organization are given in Table 2.

**Table 2:** Direct emission sources and quantities generated by the jeans-washing company in 2021

Emission sources	Amount of consumption	CO_2_e (ton/year)	CH_4_ (ton/year)	N_2_O (ton/year)	CO_2_ (ton/year)
Natural gas	612.79 t	1555.5501	1552.6148	2.1080	0.8211
Delivery vehicle (diesel)	56,144 km	14.8944	14.7900	-	0.1044
Passenger vehicle (diesel)	22,766 km	3.1321	3.0893	0.0001	0.0428
Generator (diesel)	400 lt	1.0822	1.0672	0.0001	0.0149
Forklift (diesel)	400 lt	1.0822	1.0672	0.0001	0.0149
Fire–CO_2_ tube storage	35 kg	0.0014	-	-	-

The overall amount of direct emissions related to the organization sums up to 1575.75 tCO_2_e for the year 2021

#### Indirect energy–related emissions

The emissions from the energy purchased for the organization’s activities are expressed as indirect energy–related emissions. The electricity purchased by the organization is produced from fossil fuel energy sources. Therefore, electricity consumption has a share in the carbon footprint. Total electricity consumption accounted for 1,618,396 kWh in 2021. The emission factor was determined by applying the national grid average emission factor that best characterizes the relevant grid. This emission factor has been calculated according to the data in the report published by the Turkish Electricity Generation Corporation (EÜAŞ 2021). The primary calculating method is the multiplication of consumption data and relevant EFs. The total amount of emissions related to the electricity the organization uses adds up to 798.09 t of CO_2_e for 2021.

#### Indirect non-energy–related emissions

The company generates a lot of waste as a result of many activities. The wastes generated are stored in the waste site and given to licensed companies for disposal and recycling. Domestic waste, paperboard, plastics, metals, paper packaging, batteries and accumulators, oils, and hazardous waste are all considered waste fractions. The produced waste in tonnes per year was used to determine the emissions. The amount of waste multiplied by emission factors, which indicate the amount of CO_2_ equivalent per kg of waste and were obtained from the DEFRA emission database according to waste characteristics, generated the waste-related carbon footprint. A total of 142.60 t wastes were generated in this organization in 2021. Further detailed information about the collected waste data and the total amount of greenhouse gas emissions generated is presented in Table 3.

**Table 3:** Indirect emissions generated by waste in the denim-washing company in 2021

Type of waste	Amount of waste	Emissions of carbon dioxide
	t	tCO_2_e
Domestic waste	40.193	17.93578
Sludges from on-site wastewater treatment (combustion)	20.250	0.43119
Sludges from on-site wastewater treatment (landfill)	69.800	32.59980
Processed textile fiber waste	0.339	0.15833
Waste print toners containing hazardous materials	0.007	0.00327
Engine, gearbox, and lubricating oils	0.025	0.00053
Paper and cardboard packaging	0.080	0.00170
Plastic packaging	4.880	0.10391
Wooden packaging	0.040	0.00085
Metallic packaging	0.470	0.01001
Packaging containing residues of hazardous substances or contaminated with hazardous substances	2.319	1.08308
Absorbers contaminated with hazardous substances, filter materials, cleaning cloths, protective clothing	4.020	1.87752
Gases in pressure tanks	0.005	0.00011
Laboratory chemicals consisting of or containing hazardous substances, including mixtures of laboratory chemicals	0.004	0.00009
Permanganate (e.g., potassium permanganate)	0.040	0.00085
Wastes whose collection and disposal are subject to special treatment to prevent infection	0.004	0.00009
Fluorescent lamps and other mercury-containing waste	0.020	0.00934
Oils and fats	0.100	0.04670
Batteries and accumulators	0.001	0.00002

The emissions related to sludges from on-site wastewater treatment to landfill currently have the highest emissions, as they account for around 60.08% of the total carbon footprint from waste. Those emissions are followed by domestic waste generation, with a share of about one-third total carbon footprint of organizational waste. The remaining type of waste is responsible for less than 3% of all emissions, with insignificant contribution from indirect emissions to the carbon balance. In total, the company’s waste generated 54.26 tCO_2_e (2.19% of the CF overall) in 2021.

Business travel includes work-related travel undertaken by employees of the organization, including modes of transport: buses, personal vehicles, and minibus. In addition, the facility uses rental cars and 2 buses, for personnel transportation, apart from its own vehicles. Questionnaires conducted through in-staff member interviews were used to identify the type of vehicles to be used in 2021. The types of vehicles used by transportation in 2021 and their usage percentages are given in Table 4.

**Table 4:** Results regarding a commuting survey performed at the denim-washing company

Mode of transportation	Distance traveled (km)	Percentage of usage (%)
Personal vehicle	94,800	18
Public transport	303,000	13
Company leased bus	33,088	69

**Fig. 3:**
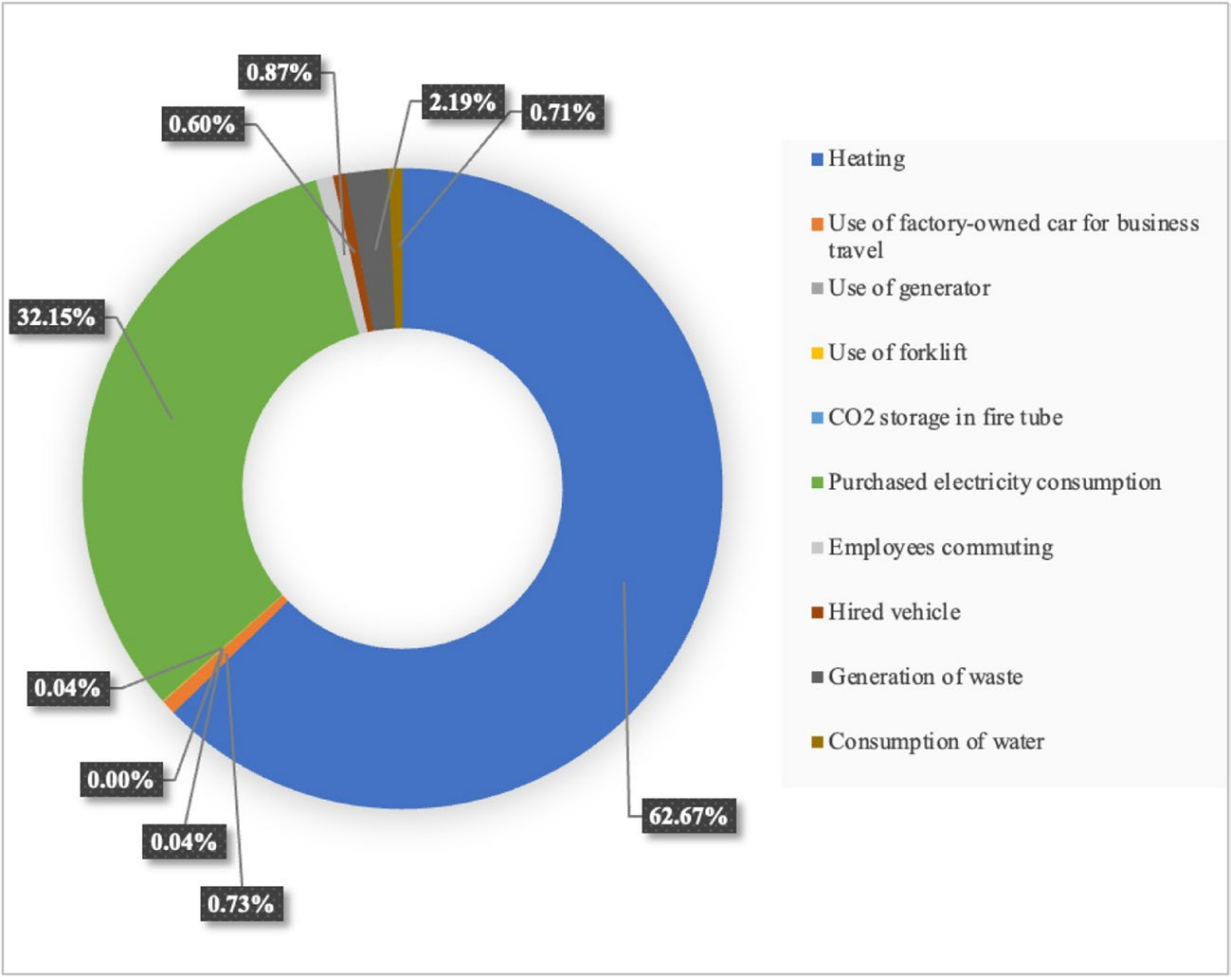
Percentages of carbon footprint by the activity of the denim-washing company in 2021

The transportation-related carbon footprint was determined by multiplying the vehicle mileage by emission factors, which represented the amount of CO_2_ equivalent per kilometer and were chosen from the DEFRA emission database based on vehicle characteristics. Personal automobiles, public transport, and rental cars constitute a remarkable portion of the total CF balance. Overall, the greenhouse gas emissions related to travel are equal to 36.404 tCO_2_e for 2021 in the scope of indirect energy–related emissions. The water consumed in the facility is considered an indirect emission. The carbon footprint due to water consumption is calculated by assuming the volume of water used in a clean water supply. In this facility, water consumption for 2021 is 118,000 m^3^. To calculate GHG emissions from water consumption, the water supplied through the network was considered in m^3^, and the water source conversion factor from the DEFRA database was used. In that regard, the factory’s carbon footprint for the consumed water in 2021 corresponds to 17.582 t/CO_2_e. The main contributions of activities as direct and indirect energy–related and indirect non-energy–related categories of the organizational carbon footprint of the jeans washing company are shown in Fig. 3. The overall quantity of emissions caused by the denim-washing company sums up to 2482.09 t CO_2_e for the year 2021. Natural gas consumption has the highest effect because it contributes to about 62.67% of the carbon footprint overall. A recent study also indicates that emissions from natural gas for heating have the most significant quantity of emissions when measured in terms of CF in the textile industry (Başoğul et al. 2021). Natural gas consumption is followed by emissions associated with electricity (32.15%). Electricity consumption accounts for the second largest share of the overall organizational carbon footprint in the denim-washing company, which is a result of the operation of the processes, machinery, and infrastructure in the buildings that belong to the company. A recent review of the literature on this topic was consistent with the results of this study (Huang et al. 2017; Radonjič and Tompa 2018). Purchased electricity is one of the main sources of greenhouse gas emissions for many companies. For example, Radonjič and Tompa confirmed that the largest contributor to greenhouse gas emissions is the consumption of purchased electricity in their organizational CP study (Radonjič and Tompa 2018). Huang and coworkers presented a detailed analysis of the Chinese textile industry and reported that the main source of greenhouse gas emission in the textile industry is primary resources, which correspond to 80% of the total emissions in the Chinese textile industry, and the second largest greenhouse gas emission source stems from electric consumption (Huang et al. 2017). Generation of waste by the denim-washing company has a lower share than heating and electricity, but with around 2.19%, it is still significant. Employees commuting, using factory-owned cars, consumption of water, and renting cars account for about 0.87%, 0.73%, 0.71%, and 0.60%, respectively. The remaining categories are much smaller and appear to constitute an almost negligible percentage. These outcomes are consistent with the context reported by Kiehle et al. and Radonjič (Kiehle et al. 2023; Radonjič and Tompa 2018). Also, Amanthi and Navarante confirmed that their company had a minimal impact on water usage, which accounts for 0.3% of the total (Awanthi and Navaratne 2018).

**Fig. 4:**
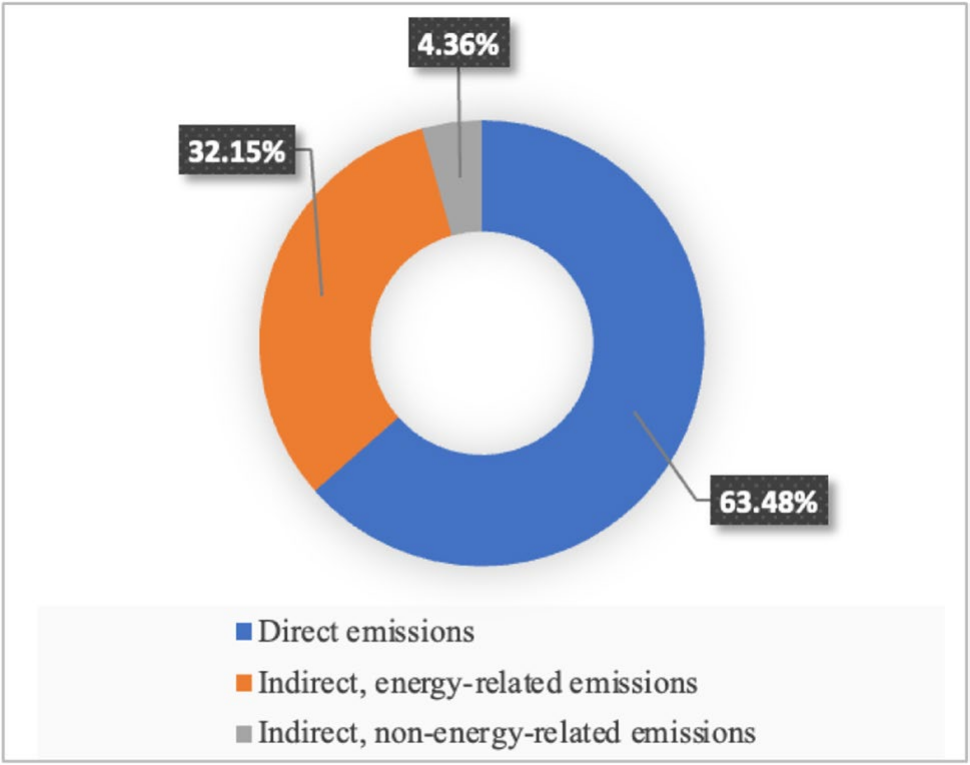
Calculated CF of the organization under three categories

The study was also examined according to operational scopes, and their contribution to CF is shown in Fig. 4 as percentages. The carbon footprint is 63.48% (1575.75 tCO_2_e/year), with significant emissions coming from direct emissions, including heating and fuel consumption. In particular, natural gas for heating plays a critical role in this organization’s greenhouse gas emissions caused by human activity. The contribution purchased electricity consumption categorized as indirect energy–related emission sources to the overall organizational CF represents almost 32.15% (798.09 tCO_2_e/year). The organization’s lowest environmental impact comes from its indirect non-energy–related carbon footprint, which makes up 4.36% (108.25 tCO_2_e/year) of the overall CF. The complex operations carried out by the organization are what cause the lowest CF value for indirect non-energy–related emission sources such as employees commuting, rental vehicles, generation of waste, and consumption of water.

In summary, this article discusses the equivalent amount of carbon dioxide produced by a denim-washing company’s own activities based on ISO standard calculation methods of greenhouse gas emissions. The most significant contributor to greenhouse gas emissions is direct emission, which is the consumption of natural gas used for heating. This is followed by purchased electricity consumption categorized as indirect energy–related emission. This study also analyzed emissions, including employee travel, rental vehicles, water consumption, and waste generation in order to understand the impact of non-energy-related indirect emissions that are barely addressed in academic studies.

### Strategies to mitigate the carbon footprint of the denim company

The determination of the organization’s carbon footprint can serve as an important identification tool for reducing greenhouse gas emissions and the implementation of a policy of low/carbon neutral products. This section deals with aspects of the reduction of organizational carbon emissions for denim-washing textile companies. As previously stated, the heating needed for the building is the main source of emissions in this case study. It is essential to focus on minimizing this topic. Perhaps it would be best to investigate more effective heating options with lower carbon footprints. In recent years, many studies have been carried out on the use of renewable energy sources, mainly hydrogen, as an alternative fuel for heating purposes (Dodds et al. 2015; Momirlan and Veziroglu 2002). Fuel cells and hydrogen can potentially generate low-carbon heat and electricity (Niknam et al. 2013), so it is possible to reduce the carbon footprint in the denim industry (Nicoletti et al. 2015, Padro and Putsche 1999).

As it is known, the textile industry is one of the sectors with the highest energy use, as seen in this case study. The most effective way to reduce energy use in the denim-washing sector is to establish energy-saving policies (Larsen et al. 2013). To optimize energy savings in a company, it is necessary to raise awareness, increase knowledge, and ensure the cooperation of everyone involved in the production process (Ozturk 2005). Another way is that the greenhouse gas emission component can be anticipated to decline if the source of electricity can be changed to more sustainable energy sources, such as renewable energy (Huang et al. 2017). In other words, ways to reduce the power consumed must be found to make the operation more environmentally friendly. An important aspect that needs to be evaluated is the waste generated from activities as it has the third highest impact on the total carbon footprint in this study. One of the most promising approaches for reducing the carbon footprint in the denim industry is recycling (Muthu et al. 2012). Paper, plastic products, metals, textiles, organic waste, batteries, and electronic waste can be reused. It is stated that by implementing the recycling and reuse process for end-of-life products, a maximum reduction of 12 kg CO_2_ equivalent per kg of textile product can be achieved (Payet 2021). It has been noted that the use of textile wastes as insulation reduces the material’s heat transfer coefficient. This kind of waste recycling will support both energy conservation and the insulation sector (Hadded et al. 2016; Islam and Bhat 2019). Travel policies play an important role in its efforts to reduce greenhouse gas emissions for this case study. A surprisingly considerable share of the case organization’s carbon footprint was due to the means of travel used by this company. The company should receive encouragement and support to choose more sustainable ways of business travel and establish a travel policy (Ozawa-Meida et al. 2013). To reduce its travel-related carbon footprint, this company should avoid unnecessary travel, conduct online meetings using video conferencing and other digital technologies, reduce car trips, travel more by train or intercity bus, and create more opportunities to work from home (El Geneidy et al. 2021; Sangwan et al. 2018).

## Conclusion

By assessing the carbon footprint of a denim-washing company, the most significant contributor to greenhouse gas emissions is direct emission, which is the consumption of natural gas used for heating. This is followed by purchased electricity consumption categorized as indirect energy–related emission. The determination of the carbon footprint of a denim-washing facility where fossil fuel use and electricity consumption are high can contribute to improving the environmental performance of the organization. Identification of greenhouse gas activities and calculation of emission amounts become very important in determining the effects of companies on climate change while carrying out their activities. It is possible to compare the carbon footprint calculated annually in the company with the values calculated every year. Carbon footprint calculation will enable the organization to document and archive the organization’s information regularly with the inventory study of the organization. In addition, accuracy checks on the data will be possible. Information management processes will be improved through periodic internal audits and technical reviews. The organization will be able to establish activities at the organizational level to reduce greenhouse gas emissions or increase greenhouse gas removals. The management of electricity consumption and utilization, energy effectiveness, technological process and development, management of travel demand, afforestation, waste minimization, use of alternative energy sources and raw materials to avoid waste disposal or incineration, and refrigerant regulation are a few examples of these activities.

As a result, calculating the carbon footprint of organizations will increase the brand image by providing positive feedback in terms of climate and environmentally sensitive stance. By having their carbon footprint calculated, institutions will be able to use it quite effectively as a marketing strategy. In addition, low-carbon/carbon–neutral products and services will also gain value rapidly from customers and investors, making these products the reason for preference.

## Data Availability

The data that support the findings of this study are available from the corresponding author on reasonable request.

## References

[CR1] Alvarez S, Rubio A (2015). Carbon footprint in Green Public Procurement: a case study in the services sector. J Clean Prod.

[CR2] Awanthi M, Navaratne C (2018). Carbon footprint of an organization: a tool for monitoring impacts on global warming. Procedia Eng.

[CR3] Başoğul Y, Göksu TT, Baran MF (2021) A case study on the assessment of carbon footprint of a textile factory. Eur J Sci Tech 31:146–150

[CR4] Cagiao J, Gómez B, Doménech JL, Mainar SG, Lanza HG (2011). Calculation of the corporate carbon footprint of the cement industry by the application of MC3 methodology. Ecol Ind.

[CR5] Chang J, Zhang H, Wang Y, Zhu Y (2015). Assessing the impact of climate variability and human activity to streamflow variation. Hydrol Earth Syst Sci Discuss.

[CR6] Dawkins C, Fraas JW (2011). Coming clean: the impact of environmental performance and visibility on corporate climate change disclosure. J Bus Ethics.

[CR7] DEFRA (2021) Department for Environment Food & Rural Affairs, Conversion Factor. https://www.gov.uk/government/publications/greenhouse-gas-reporting-conversion-factors-2021. Accessed 13 March 2022

[CR8] Dodds PE, Staffell I, Hawkes AD, Li F, Grünewald P, McDowall W, Ekins P (2015). Hydrogen and fuel cell technologies for heating: a review. Int J Hydrogen Energy.

[CR9] El Geneidy S, Baumeister S, Govigli VM, Orfanidou T, Wallius V (2021). The carbon footprint of a knowledge organization and emission scenarios for a post-COVID-19 world. Environ Impact Assess Rev.

[CR10] Eslamidoost Z, Arabzadeh M, Oskoie V, Dehghani S (2022). Carbon footprint calculation in one of the largest gas refinery companies in the Middle East. Environ Sci Pollut Res.

[CR11] UNFCCC (2016) The Paris Agreement. https://unfccc.int/process-and-meetings/the-paris-agreement. Accessed 15 Jan 2022

[CR12] EUAS (2021) Electricity generation in Turkey, Annual Report.https://www.euas.gov.tr/en-US. Accessed 17 Jan 2022

[CR13] Foran B, Lenzen M, Dey C, Bilek M (2005). Integrating sustainable chain management with triple bottom line accounting. Ecol Econ.

[CR14] Gao T, Liu Q, Wang J (2014). A comparative study of carbon footprint and assessment standards. Int J Low-Carbon Technol.

[CR15] Hadded A, Benltoufa S, Fayala F, Jemni A (2016). Thermo physical characterisation of recycled textile materials used for building insulating. J Build Eng.

[CR16] Harangozo G, Szigeti C (2017). Corporate carbon footprint analysis in practice–with a special focus on validity and reliability issues. J Clean Prod.

[CR17] Huang B, Zhao J, Geng Y, Tian Y, Jiang P (2017). Energy-related GHG emissions of the textile industry in China. Resour Conserv Recycl.

[CR18] Islam S, Bhat G (2019). Environmentally-friendly thermal and acoustic insulation materials from recycled textiles. J Environ Manag.

[CR19] International Organization for Standardization (2006) ISO 14064-1: 2018 Greenhouse Gases–Part 1: Specification with Guidance at the Organization Level for Quantification and Reporting of Greenhouse Gas Emissions and Removals

[CR20] Karalis D, Kanakoudis V (2023). Carbon footprint of products and services: the case of a winery in Greece. Sci Total Environ.

[CR21] Khan S, Malik A (2014) Environmental and health effects of textile industry wastewater. In: Malik A, Grohmann E, Akhtar R (eds) Environmental Deterioration and Human Health. Springer, Dordrecht pp 55–71

[CR22] Kiehle J, Kopsakangas-Savolainen M, Hilli M, Pongrácz E (2023). Carbon footprint at institutions of higher education: the case of the University of Oulu. J Environ Manag.

[CR23] Kishor R, Purchase D, Ferreira L, Mulla S, Bilal M, Bharagava R (2020) Environmental and health hazards of textile industry wastewater pollutants and its treatment approaches. In: Hussain C (ed) Handbook of Environmental Materials Management Cham, Switzerland Springer Nature pp 1–24

[CR24] Larsen HN, Pettersen J, Solli C, Hertwich EG (2013). Investigating the carbon footprint of a university-the case of NTNU. J Clean Prod.

[CR25] Lee KH, Cheong IM (2011) Measuring a carbon footprint and environmental practice: the case of Hyundai Motors Co.(HMC). Ind Manage Data Syst 111:961–978

[CR26] Matthews HS, Hendrickson CT, Weber CL (2008) The importance of carbon footprint estimation boundaries. Environ Sci Technol 42:5839–5842 10.1021/es703112w 18767634

[CR27] Momirlan M, Veziroglu T (2002). Current status of hydrogen energy. Renew Sustain Energy Rev.

[CR28] Muthu SS, Li Y, Hu JY, Ze L (2012). Carbon footprint reduction in the textile process chain: recycling of textile materials. Fibers Polym.

[CR29] Nicoletti G, Arcuri N, Nicoletti G, Bruno R (2015). A technical and environmental comparison between hydrogen and some fossil fuels. Energy Convers Manag.

[CR30] Niknam T, Bornapour M, Gheisari A (2013). Combined heat, power and hydrogen production optimal planning of fuel cell power plants in distribution networks. Energy Convers Manag.

[CR31] Ozawa-Meida L, Brockway P, Letten K, Davies J, Fleming P (2013). Measuring carbon performance in a UK university through a consumption-based carbon footprint: De Montfort University case study. J Clean Prod.

[CR32] Ozturk HK (2005). Energy usage and cost in textile industry: a case study for Turkey. Energy.

[CR33] Padro CE, Putsche V (1999) Survey of the economics of hydrogen technologies, Technical Report (No. NREL/TP-570-27079), National Renewable Energy Lab. (NREL), Golden, CO, United States

[CR34] Padrón RS, Gudmundsson L, Decharme B, Ducharne A, Lawrence DM, Mao J, Peano D, Krinner G, Kim H, Seneviratne SI (2020). Observed changes in dry-season water availability attributed to human-induced climate change. Nat Geosci.

[CR35] Payet J (2021). Assessment of carbon footprint for the textile sector in France. Sustainability.

[CR36] Peters GP (2010). Carbon footprints and embodied carbon at multiple scales. Curr Opin Environ Sustain.

[CR37] Radonjič G, Tompa S (2018). Carbon footprint calculation in telecommunications companies–the importance and relevance of scope 3 greenhouse gases emissions. Renew Sustain Energy Rev.

[CR38] Robinson OJ, Tewkesbury A, Kemp S, Williams ID (2018). Towards a universal carbon footprint standard: a case study of carbon management at universities. J Clean Prod.

[CR39] Ruževičius J, Dapkus M (2018). Methodologies for calculating the carbon footprint of small organizations. Calitatea.

[CR40] Saenz J, Figliozzi M, Faulin J (2016). Assessment of the carbon footprint reductions of tricycle logistics services. Transp Res Rec.

[CR41] Sangwan KS, Bhakar V, Arora V, Solanki P (2018). Measuring carbon footprint of an Indian university using life cycle assessment. Procedia CIRP.

[CR42] Scipioni A, Manzardo A, Mazzi A, Mastrobuono M (2012). Monitoring the carbon footprint of products: a methodological proposal. J Clean Prod.

[CR43] Sivaram N, Gopal P, Barik D (2019) Toxic waste from textile industries, in: Energy From Toxic Organic Waste for Heat and Power Generation, Elsevier pp 43–54

[CR44] Sundarakani B, De Souza R, Goh M, Wagner SM, Manikandan S (2010). Modeling carbon footprints across the supply chain. Int J Prod Econ.

[CR45] Weidema BP, Thrane M, Christensen P, Schmidt J, Løkke S (2008). Carbon footprint: a catalyst for life cycle assessment?. J Ind Ecol.

[CR46] Wiedmann T, Minx J (2008). A definition of ‘carbon footprint’. Ecol Econ Res Trends.

[CR47] Xie S, Mo X, Hu S, Liu S (2020). Contributions of climate change, elevated atmospheric CO2 and human activities to ET and GPP trends in the Three-North Region of China. Agric For Meteorol.

[CR48] Yan Y, Wang C, Ding D, Zhang Y, Wu G, Wang L, Liu X, Du C, Zhang Y, Zhao C (2016). Industrial carbon footprint of several typical Chinese textile fabrics. Acta Ecol Sin.

[CR49] Ye X, Zhang Q, Liu J, Li X, Xu C-y (2013). Distinguishing the relative impacts of climate change and human activities on variation of streamflow in the Poyang Lake catchment, China. J Hydrol.

[CR50] Zhang Y, Wang X, Qin S (2013). Carbon stocks and dynamics in the three-north protection forest program, China. Austrian J For Sci.

[CR51] Zhou Q, Le QV, Meng L, Yang H, Gu H, Yang Y, Chen X, Lam SS, Sonne C, Peng W (2022). Environmental perspectives of textile waste, environmental pollution and recycling. Environ Technol Rev.

